# Exercise therapy and cognitive behavioural therapy to improve fatigue, daily activity performance and quality of life in Postpoliomyelitis Syndrome: the protocol of the FACTS-2-PPS trial

**DOI:** 10.1186/1471-2377-10-8

**Published:** 2010-01-18

**Authors:** Fieke S Koopman, Anita Beelen, Karin H Gerrits, Gijs Bleijenberg, Tineke A Abma, Marianne de Visser, Frans Nollet

**Affiliations:** 1 Department of Rehabilitation, Academic Medical Centre, University of Amsterdam, Amsterdam, The Netherlands; 2 Research Institute MOVE, Faculty of Human Movement Sciences, VU University Amsterdam, Amsterdam, The Netherlands; 3 Expert Centre Chronic Fatigue Nijmegen, Radboud University Nijmegen Medical Centre, Nijmegen, The Netherlands; 4 Department of Medical Humanities, VU University Medical Centre, Amsterdam, The Netherlands; 5 Department of Neurology, Academic Medical Centre, University of Amsterdam, Amsterdam, The Netherlands

## Abstract

**Background:**

Postpoliomyelitis Syndrome (PPS) is a complex of late onset neuromuscular symptoms with new or increased muscle weakness and muscle fatigability as key symptoms. Main clinical complaints are severe fatigue, deterioration in functional abilities and health related quality of life. Rehabilitation management is the mainstay of treatment. Two different therapeutic interventions may be prescribed (1) exercise therapy or (2) cognitive behavioural therapy (CBT). However, the evidence on the effectiveness of both interventions is limited. The primary aim of the FACTS-2-PPS trial is to study the efficacy of exercise therapy and CBT for reducing fatigue and improving activities and quality of life in patients with PPS. Additionally, the working mechanisms, patients' and therapists' expectations of and experiences with both interventions and cost-effectiveness will be evaluated.

**Methods/Design:**

A multi-centre, single-blinded, randomized controlled trial will be conducted. A sample of 81 severely fatigued patients with PPS will be recruited from 3 different university hospitals and their affiliate rehabilitation centres. Patients will be randomized to one of three groups i.e. (1) exercise therapy + usual care, (2) CBT + usual care, (3) usual care. At baseline, immediately post-intervention and at 3- and 6-months follow-up, fatigue, activities, quality of life and secondary outcomes will be assessed. Costs will be based on a cost questionnaire, and statistical analyses on GEE (generalized estimated equations). Analysis will also consider mechanisms of change during therapy. A responsive evaluation will be conducted to monitor the implementation process and to investigate the perspectives of patients and therapists on both interventions.

**Discussion:**

A major strength of the FACTS-2-PPS study is the use of a mixed methods design in which a responsive and economic evaluation runs parallel to the trial. The results of this study will generate new evidence for the rehabilitation treatment of persons with PPS.

**Trial registration:**

Dutch Trial Register NTR1371.

## Background

Poliomyelitis anterior acuta is an acute viral disease that attacks the anterior horn cells of the spinal cord and the motor neurons of the lower brain stem resulting in flaccid paresis or paralysis. Usually there is partial and sometimes complete recovery from the self-terminating disease. However, many people with a history of poliomyelitis report late onset neuromuscular symptoms and a decline in functional abilities. These late symptoms are referred to as Postpoliomyelitis Syndrome (PPS) and include new or increased muscle weakness, abnormal muscle fatigability, generalized fatigue, muscle atrophy, muscle and joint pain, muscle cramps and cold intolerance [1].

The prevalence of PPS has been reported from 15% to 80% of all patients with previous paralytic polio depending on the criteria applied and population studied [2]. Nearly 60% of a sample of Dutch survivors of the 1956 polio outbreak experience late onset polio sequelae [3]. In Western countries, where the large epidemics date back to the 1940s and 1950s, many polio survivors are now experiencing progressive complaints related to PPS. The World Health Organization (WHO) estimates that 10 to 20 million polio survivors are alive worldwide, and some estimates suggest that 4 to 8 million of them may develop PPS [4]. Although, the efforts of the Global Polio Eradication Initiative of the WHO initiated in 1988, led to an enormous reduction in the number of acute polio cases globally ever since, polio is still a relevant problem. In Western Africa and in South Asia polio is still endemic and spread of the virus from these countries causes new outbreaks in countries that were certified as polio free. Therefore, new cases of PPS can be anticipated in the coming decades.

Fatigue is one of the most frequent complaints of PPS [5-7] and it is typically described as tiredness or lack of energy that increases with physical activity and decreases with rest [8]. In a study on disability and health problems in 76 Dutch patients with PPS, 78% of the subjects selected fatigue as their major problem [9]. Subjects with PPS experience higher levels of fatigue than healthy controls [10,11]. Fatigue has a negative impact on activities of daily living and there is evidence that post-polio related fatigue is an important factor for the reduced health related quality of life (HRQoL) in polio survivors [12].

In current practice, rehabilitation management is the mainstay of treatment for PPS. Rehabilitation aims to improve patients' capacities to perform activities of daily living and adapt performance (i.e. actual behaviour) to the available capacities. We expect that a reduction of the imbalance between patients' capacities and performance will lead to a reduction in fatigue and improvement in activities and HRQoL. There are two possible approaches to achieve this goal; exercise therapy and cognitive behavioural therapy (CBT). However, evidence to support the effectiveness of either approach is still limited.

The insufficient evidence to support exercise consists of contradictory and incomplete information in the literature. On the one hand, PPS patients are advised to avoid muscular overuse and intensive training as this could worsen symptoms such as muscle weakness and fatigue and provoke a further loss of muscular strength [1]. On the other hand, physically active PPS patients were found to have less symptoms of fatigue than sedentary patients [13]. It is unclear whether symptoms of fatigue are cause or result of physical inactivity. A systematic review on exercise therapy for neuromuscular diseases included ten studies on muscle strengthening exercises and aerobic exercises in PPS of which five demonstrated significant positive effects on muscular strength and aerobic capacity without generating any adverse effects. However, all these studies had to be qualified as having insufficient or limited methodological quality [14]. This led to the conclusion that there is insufficient evidence for the effectiveness of exercise for patients with PPS and that future, preferably multi-centre, studies are needed. A recent study on the short-term effectiveness of home- and hospital- based aerobic exercise showed improvement on fatigue and quality of life [15]. However results were not compared with a control group and long-term effectiveness was not evaluated in this study.

There is broad evidence for the effectiveness of CBT in reducing fatigue in chronic fatigue syndrome, fatigued post cancer survivors and multiple sclerosis [16-19]. However, there are no studies of CBT for fatigue in PPS.

The evidence for a cognitive behavioural approach in PPS is currently limited to results from an uncontrolled pilot study in which cognitive behavioural strategies are incorporated in a comprehensive multidisciplinary rehabilitation program [20]. Although significant reductions in fatigue symptoms were found, it is unclear whether these effects can be ascribed to the cognitive behavioural components of the intervention.

We anticipate that exercise therapy and CBT are both effective in reducing fatigue and improving activities and HRQoL in patients with PPS compared to the usual care. The FACTS-2-PPS study aims to give insight in the efficacy of both interventions for patients with PPS. As secondary outcomes, the working mechanisms, patients' and therapists' expectations of and experiences with both interventions and cost-effectiveness will be evaluated. These aims have led to the following research questions:

(1) Does exercise therapy for patients with PPS reduce fatigue and improve activities and HRQoL as compared to usual care?

(2) Does CBT for patients with PPS reduce fatigue and improve activities and HRQoL as compared to usual care?

(3) What are the generic and disease-specific determinants for treatment success of exercise therapy and CBT?

(4) What are patients' expectations of and experiences with exercise therapy and CBT?

(5) What is the cost-effectiveness of exercise therapy and CBT compared to usual care?

## Methods/Design

### Study design

**Figure 1:**
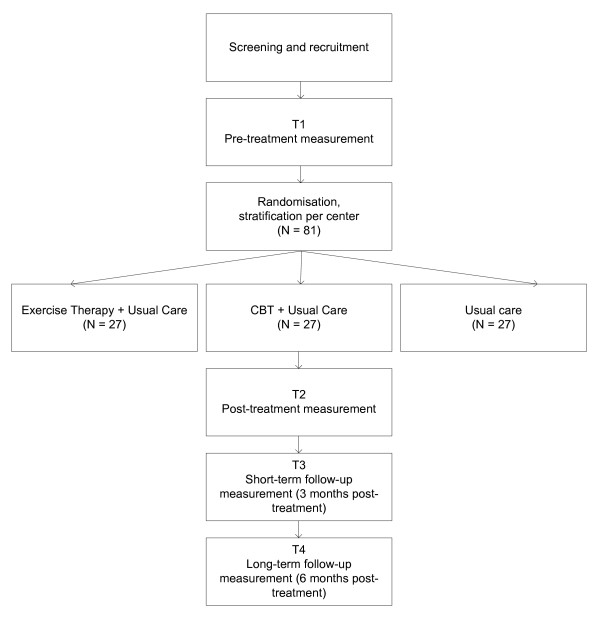
**Study design**.

A multi-centre, single-blinded randomized controlled trial (RCT) with 6 months follow-up will be conducted to evaluate the efficacy of exercise therapy and CBT compared to usual care in patients with PPS (Figure 1). To gain insight into patients' expectations and experiences, a responsive evaluation will be conducted. To determine the cost-effectiveness, an economic evaluation will be conducted alongside this RCT. The study protocol was approved by the Medical Ethics Committee of the Academic Medical Centre (AMC) and all participating centres granted approval to participate.

### Study population

All patients will be recruited from outpatient clinics of the AMC Amsterdam; the University Medical Centre Utrecht (UMCU); the University Medical Centre Nijmegen (UMCN) or the affiliate rehabilitation centres. Case histories are checked to screen for potentially eligible patients. Patients willing to give signed consent are evaluated by a physician to check in and exclusion criteria (Table 1). The sample size was calculated for this three-arm trial based on the comparison treatment (exercise therapy or CBT) versus usual care, with equal allocation across treatment arms and 4 repeated measurements (at entry (pre-treatment), at 4 months (post-treatment), at 3 months follow-up (short-term follow-up) and at 6 months follow-up (long-term follow-up), respectively), with an estimated correlation coefficient of the repeated measurements of rho = 0.79 (based on unpublished data from a reproducibility study in 37 PPS patients), a clinical relevant improvement of 8 points on the CIS-fatigue scale (for both treatments) [17] and an estimated standard deviation in each treatment group of 9.3. The total sample size needed to detect this difference on a 5% level of significance (two tailed) with a power of 90% was 24 subjects in each group [21]. We expect a drop-out rate of maximal 10%, based on a previous trial in this patient group [22]. Therefore 81 patients will be recruited.

**Table 1:** In- and exclusion criteria

Inclusion criteria
(1) diagnosis of PPS according to the criteria of March of Dimes [1]

(2) severe perceived fatigue (CIS-fatigue > = 35) [47]

(3) age between 18 and 75 years

(4) life-expectancy longer than one year

(5) walking-ability at least indoors with or without a walking aid

(6) ability to cycle on a cycle ergometer against a load of at least 25 Watt

**Exclusion criteria**

(1) use of psychotropic drugs or other psychiatric treatment

(2) clinical depression (BDI-PC > 6) [48]

(3) disabling co-morbidity interfering with the intervention programs or influencing outcome parameters (including cardiopulmonary disease, epileptic seizures, poorly regulated diabetes mellitus)

(4) respiratory insufficiency (FVC < 50%pred or CO_2 _retention) or assisted ventilation

(5) cognitive impairment

(6) insufficient mastery of the Dutch language

(7) pregnancy

### Randomization and blinding

Patients will be assigned to the rehabilitation centre that is nearest to their home village. Randomization will be stratified per centre. Patients fulfilling both inclusion and exclusion criteria will be randomized to one of three groups: (1) exercise therapy + usual care, (2) CBT + usual care, or (3) usual care. The randomization scheme is computer-generated and will be done by creating random blocks of sequences with variable block sizes of 3 and 6. The investigator who will perform the randomization will inform the patient and, in case of allocation to exercise therapy or CBT also the therapist of the group allocation.

The investigators that are responsible for the inclusion will be blinded. Outcomes will be assessed by blinded and independent outcome assessors. At the beginning of the assessment patients will be instructed not to reveal their group allocation to the investigators. To evaluate the success of blinding, the investigator will be asked at each measurement, to guess the treatment assignment of the patient, with options 'CBT', 'exercise therapy', 'usual care only' and 'unknown'. Analyses will be performed blinded for treatment allocation.

### Interventions

#### Usual care

The patients in the control and intervention groups will all receive usual care. Usual care for PPS patients may include usage of assistive devices, orthoses, physical therapy, and medication. Patients will not be restricted in their activities. Co-interventions will be monitored throughout the study.

#### Exercise therapy

Exercise therapy is designed specifically to enhance physical capacity. The intervention has a duration of 16 weeks and consists of (1) a home-based aerobic training program three times weekly and a (2) supervised group training consisting of muscle strengthening and functional exercises once a week. The therapy will be supervised by specifically trained physiotherapists. An unblinded member of the project group will supervise the therapists and perform integrity checks on each treatment location.

(1) The home-based training program consists of individually tailored aerobic exercise on a cycle ergometer. Patients will be supplied with a cycle ergometer, a log book with training instructions and a training scheme at home. Heart rate will be continuously measured with a heart rate monitor. Furthermore, in the log book patients will document the number and duration of treatment sessions, the training load, perceived exertion on the Borg Rated Perceived Exertion Scale [23] and possible complaints after the training session. Training intensity will be gradually increased from 60% of the Heart Rate Reserve (HRR) to 70% HRR, in accordance with the American College of Sports Medicine guidelines for aerobic training in healthy adults [24] and persons with chronic diseases and disabilities [25]. The duration of the training sessions will gradually be increased from 20 to 30 minutes per session and sessions are divided into prescribed exercise bouts which are interspersed with short rest periods of unloaded cycling. The duration of exercise bouts will be gradually increased from 2 minutes at the start of the program to 13 minutes at the end of the program.

Warming-up and cooling-down consists of 5 minutes unloaded cycling. Feasibility of the training schemes is weekly checked by one of the therapists by reading out the heart rate monitors and checking the log books. When necessary, adjustments to the training schemes will be made.

(2) The supervised group training consists of individually tailored muscle strengthening exercises and functional exercises in 1-hour group sessions. Sessions will be divided into a 5-minute warm-up period of aerobic exercises, 30 minutes of muscle strengthening, 20 minutes functional exercises and a 5-minute cool-down period. Muscle strengthening exercise selection and dosage will be determined by the therapist at a separate visit to the treatment location before the start of the program. Muscle groups and accompanying exercises will be selected based on patients MRC-scores [26] and reported problems in daily activities. Exercises are selected based on the best expected effects on physical functioning.

Muscle groups with MRC scores less then 3 are not selected and quadriceps muscles are always selected when this muscle group has a MRC score of at least 3. Training load will be based on the 1-RM test [27]. In the first 4 weeks, training load will be 50% of 1-RM, each exercise consists of 3 sets with 2 minutes rest between a set and number of repetitions will be increased from 8 to 20 during 4 weeks. When the desired number of repetitions with the current load is reached, training load is increased in week 5 and 9 to 60 and 70% respectively, and the number of repetitions is increased in the same manner as prescribed for the first 4 weeks [28].

#### Cognitive behavioural therapy

CBT will be directed at frequently reported perpetuating factors of fatigue in slowly progressive neuromuscular disorders [29]. They may involve dysfunctional cognitions with respect to the disease itself, to pain and to fatigue [30], dysfunctional attention to pain or fatigue symptoms, deregulation of sleep [30,31], deregulation of physical, social and/or mental activities [30,31], and low social support and negative social interactions [32]. For each factor a standardized module is available as part of the intervention. Because of the variability of relevant perpetuating factors in PPS patients, therapy will be customized to each individual. To determine which modules will be necessary, each perpetuating factor will be measured with specific questionnaires (Table 2). The number of sessions will be determined by the number of modules used and will vary between 12 and 16 sessions, each with a duration of 1 hour during a 4 months period. Experienced cognitive behavioural therapists will treat the patients. The therapists will be trained in the protocol and the use of instruments to determine which module should be included in the therapy. Role-playing is an important part of this training. In the first CBT session individualized concrete behavioural goals of the therapy are formulated by the patient with help of the therapist. These goals are formulated in terms of concrete behaviours the patient wants to perform if he is not extremely fatigued anymore. Appendix 1 gives an overview of cognitive and behavioural techniques that can be used during CBT for each perpetuating factor of fatigue.

**Table 2:** Instrumentation for module selection CBT

Perpetuating factor for fatigue	Instrumentation
1. Dysfunctional cognitions with respect to the disease	Impact of Event Scale [49] Pictorial Representation of Self and Illness Measure (PRISM) [50] Illness Cognitions Questionnaire (ICQ) [51]

2. Dysfunctional cognitions with respect to pain	Medical Outcome Study 36-Item Short-Form Health Survey (SF-36; domain: pain) [52] Daily Observed Pain (DOP) [53] Pain Catastrophizing Scale (PCS) [54]

3. Dysfunctional cognitions with respect to fatigue	Self-Efficacy Scale [18] Jacobsen Fatigue Catastrophizing Scale (J-FCS) [55]

4. Dysfunctional attention to fatigue and pain	Illness Management Questionnaire (IMQ) [56]

5. Deregulation of sleep	Sickness Impact Profile (SIP; domain: sleep and rest) [57] Symptom Checklist-90 (SCL-90; domain: sleep) [58]

6. Deregulation of physical activities	Chronic Fatigue Syndrome-activity questionnaire (CFS-AQ; domain: physical activity) [59]

7. Deregulation of social activities	Medical Outcome Study 36-Item Short-Form Health Survey (SF-36; domain: social functioning) [52] Chronic Fatigue Syndrome-activity questionnaire (CFS-AQ; domain social activity) [59] Sickness Impact Profile (SIP; domain: social interaction) [57]

8. Deregulation of mental activities	Checklist Individual Strength (CIS; domain: concentration) [47] Sickness Impact Profile (SIP; domain: alertness/intellectual functioning) [57] Chronic Fatigue Syndrome-activity questionnaire (CFS-AQ; domain: mental) [59]

9. Low social support and negative social interactions	Social Support Inventory-Interactions (SSL-I_08) [60] Social Support Inventory-Discrepancies (SSL-D_08) [60]

### Compliance and attrition

Compliance will be assessed by recording the number of treatment sessions (CBT or group exercise sessions) attended and, for the patients randomized to exercise therapy, also the total time spent to aerobic exercise on the bicycle ergometer at home will be recorded in a log book.

### Outcomes

#### Efficacy and working mechanisms

Outcome measures are presented in Table 3. Our primary outcome measure is fatigue. Additionally, daily activity performance and HRQoL will be evaluated. Secondary outcomes are categorised in accordance with the International Classification of Functioning (ICF) [33] on the level of body functions, activities and participation and personal factors. As potential effect modifiers the following parameters will be studied; demographic variables, main complaints of PPS, disease severity, co-morbidity, serum creatine kinase activity and the ICF environmental factors; social support, HRQoL of the partner, coping of the partner and caregiver burden. Two secondary outcome measures on the level of body functions; cardio-respiratory fitness and neuromuscular capacity will be described in greater detail.

**Table 3:** Outcome measures and instrumentation

	Instrumentation	T1	T2	T3	T4
**Primary outcome measures**					

Fatigue	Checklist Individual Strength (CIS; domain: fatigue) [47]	X	X	X	X

Daily activity performance	Sickness Impact Profile 68 (SIP-68; domains: mobility range, mobility control, social behaviour) [61]	X	X	X	X

HRQoL	Medical Outcome Study 36-Item Short-Form Health Survey (SF-36) [52]	X	X	X	X

**Secondary outcome measures**					

***ICF: Body functions***					

Pain	Visual Analogue Scale (VAS)	X	X	X	X

Emotional states	Profile of Mood States (POMS) [62,63]	X	X	X	X

Sleep disturbances	Nottingham Health Profile (NHP; domain: sleep) [64]	X	X	X	X

Cardio-respiratory fitness	Submaximal exercise test with cycle ergometer	X	X	X	X

Neuromuscular capacity	Fixed dynamometry and electrical stimulation	X	X	X	X

***ICF: Activities/Participation***					

Physical activity level in daily life	Activity Monitor (StepWatch, Cyma, Seattle, WA) on 7 consecutive days	X	X	X	X

Perceived participation	Impact on Participation and Autonomy Questionnaire (IPA) [65]	X	X	X	X

Functional Capacity	Timed-Up-and-Go test (TUG) [66] and 2-minute walk test (2-MWT) [67]	X	X	X	X

***ICF: Personal Factors***					

Illness cognitions	Illness Cognitions Questionnaire (ICQ) [51]	X	X	X	X

Coping	Coping Inventory for Stressful Situations (CISS-21) [68]	X	X	X	X

General Self Efficacy	General Self Efficacy Scale (ALCOS-16) [69]	X	X	X	X

**Effect Modifiers**					

***General effect modifiers***					

Demographic variables (age, gender, education, ethnicity, social-economical status)		X			

PPS main complaints	Polio Problem List (PPL) [9]	X	X	X	X

PPS disease severity	Medical Research Council scale (MRC) [26]	X			

Co-morbidity	Cumulative Illness Rating Scale (CIRS) [70]	X	X	X	X

Serum creatine kinase activity	Venipuncture	X *	X	X	X

***ICF: Environmental factors***					

Social support	Social Support Inventory (SSL-D) [60]	X	X	X	X

HRQoL (partner)	Medical Outcome Study 36-Item Short-Form Health Survey (SF-36) [52]	X	X	X	X

Coping (partner)	Coping Inventory for Stressful Situations (CISS-21) [68]	X	X	X	X

Caregiver Strain (partner)	Caregiver Strain Index (CSI) [71]	X	X	X	X

**Economic evaluation**					

HRQoL	EuroQol-5D [41]	X	X	X	X

Resource use	Cost diaries	X	X	X	X

* In the exercise group two extra blood samples will be obtained at 5 and 10 weeks.

T1; pre-treatment, T2; post-treatment, T3; short-term follow-up, T4; long-term follow-up.

#### Cardio-respiratory fitness

Subjects will perform a submaximal exercise test on a cycle ergometer (Lode Corival, Groningen, The Netherlands). The test starts with unloaded pedalling for three minutes, followed by a 10 Watt increment every single minute and will be terminated if one of the following occurs: (1) achieving 80% HRR or, (2) pedalling frequency dropping below 60 rpm, or (3) not being able to continue the test for any reason. The same workload protocol will be applied at the follow-up measurements. Throughout the test, gas exchange variables (COSMED K4b^2^, Rome, Italy) and heart rate will be measured continuously. Cardio-respiratory fitness will be determined based on changes in heart rate and gas exchange variables.

#### Neuromuscular capacity

Maximal voluntary contraction (MVC) of the quadriceps muscle will be measured isokinetically between 90° and 30° knee flexion using a fixed dynamometer (Biodex System 3, New York, USA). To determine MVC, subjects will perform three maximal-effort knee extensions at an angular velocity of 60°/sec. The highest value of peak torque (Nm) will be used for the analyses.

In addition, under isometric conditions, voluntary activation (VA) will be measured with a modified superimposed stimulation technique [34]. The quadriceps muscle will be electrically stimulated transcutaneously with surface electrodes placed over the proximal and distal part of the anterior thigh and using a computer-controlled constant current stimulator (Digitimer DSH7, Welwyn garden City, UK). A triplet (pulse train of three 200 μs pulses applied at 300 Hz with a supramaximal stimulation current) will be superimposed on a 3-4s maximal isometric knee extension at 60° knee flexion. An identical stimulation will be delivered to the relaxed quadriceps muscle to evoke a resting (control) triplet. Voluntary activation will be calculated by the following formula:

Fatigability of the quadriceps muscle will be determined by fatiguing the muscle by a series of electrically stimulated contractions. The current will be reduced such that a single tetanus at 150 Hz evokes approximately 30-50% of maximal isometric strength. A series of trains of 50 Hz stimulation (duration 1000 ms, with 1000 ms between trains) will be applied for a period of 300 seconds (150 contractions in total). Recovery of fatigue will be monitored by applying the same 50 Hz train (1000 ms duration) at different times (15 sec up to 3 min) after the end of the protocol. Changes in force, maximal rate of force rise and half relaxation time will be determined. These parameters will be expressed as a percentage of the values obtained in the first contraction of the protocol to correct for differences in muscle strength.

#### Adverse events

All adverse events reported spontaneously by the participants or observed by the therapists will be recorded. All adverse events will be followed until they have abated, or until a stable situation has been reached.

#### Responsive evaluation

To gain insight into patients' and therapists' expectations of and experiences with both interventions, a responsive evaluation will be conducted [35,36]. Therapists and a subgroup of patients will be interviewed about their experiences with the intervention. The selection of respondents will be based on the criterion of ''maximum variation" [37]. The interviews are semi-structured, with open questions guided by a topic list. In order to check the topic list three pilot interviews will be conducted with patients and one with a partner. Then patients engaged in both interventions will be interviewed. Additionally focus groups will be organized to validate, deepen and broaden the issues in the interviews, starting with two homogeneous focus groups (converging interests) with patients and partners, and two homogenous focus groups with therapists (one per intervention). Then all parties will be brought together in a heterogeneous (diverging interests) dialogue group to exchange views. In the cyclic process data from earlier phases will be used as input for the next phase; this hermeneutic dialectic interaction prevents one-sidedness and fosters the validation of data [38]. During all phases the interviews and focus groups will be tape-recorded, transcribed and analyzed. The analysis will focus on the recurring issues and concerns of all parties, and the comparison of perspectives. Member checks will be held to check the credibility of the analysis [37]. In order to consult and gain advice from patient representatives with PPS the research team will collaborate with the Vereniging Spierziekten Nederland (Dutch patient support group), in all phases of the research process [39].

#### Economic evaluation

Cost-effectiveness and cost-utility will be evaluated from a societal perspective. The costs will include direct health care costs of visits to general practitioners, medical specialists, therapists, medications and assistive devices, direct non-health care costs i.e. non-reimbursable costs for complementary medicine and over-the-counter medication, and indirect, non-health care costs of changes in paid and unpaid work. All these data will be collected via cost diaries.

Resource use will be valued following the procedures outlined by the Dutch Manual of Costing [40]. Where standard cost prices are not available, tariffs or professional fees will be used to estimate costs. The costs of medications will be estimated using the prices reported by the Royal Dutch Society for Pharmacy. Productivity loss costs from paid work will be estimated using both the human capital approach and friction cost method. The productivity loss costs from unpaid work will be determined using a shadow price. Cost prices of the interventions will be determined by a bottom-up calculation.

The effect measures used in the economic evaluation will be our primary outcome measure fatigue and HRQoL measured with the EuroQol-5D [41] at the four different time measurements. Utilities will be derived using the Dutch tariffs and used to determine quality-adjusted life years (QALYs) [42,43].

### Statistical Analyses

GEE (generalized estimated equations) analyses will be used to investigate differences in the effects (primary and secondary outcomes) between both intervention groups and the usual care group and to investigate associations between potential effect modifiers and effect of interventions. Data will be analyzed according to the intention-to-treat principle. Missing data will be imputed by carrying the last observation forward.

The economic evaluation will also be performed according to the intention-to-treat principle. First, mean differences in QALYs will be tested using parametric tests, and uncertainty expressed by 95% confidence intervals. Second, we will conduct between-group comparisons of the mean costs for each of the resource use categories and total costs. Confidence intervals around the mean cost differences will be obtained by a bias-corrected and accelerated (Bca) bootstrapping based on 5000 replications [44]. Third, the incremental cost-effectiveness ratio (ICER) will be determined by dividing the mean difference in total costs by the mean difference in effects. Insight into the uncertainty around the mean ICER will be obtained by bootstrapping based on 5000 replications, the generation of cost-effectiveness planes [45] and cost-effectiveness acceptability curves [46]. Sensitivity analyses involving the most important cost drivers will be performed in order to assess the robustness of the results.

## Discussion

In the FACTS-2-PPS study, the efficacy of exercise therapy and CBT in reducing fatigue and improving activities and HRQoL in patients with PPS will be evaluated in comparison with usual care. This study captures some important strengths.

Firstly, the majority of studies on exercise in PPS evaluated the effects of strength or aerobic training in PPS separately. However, to execute normal every day activities, more integrated functional tasks are necessary requiring a combination of strength, endurance, flexibility, balance and coordination. This study evaluates a multi-component exercise training in which aerobic, strength and functional exercises are combined.

Secondly, although it is widely recommended that behavioural adaptation is an important component in the management of fatigue in PPS, there are no studies confirming the effectiveness of this approach. This is the first study that will evaluate the efficacy of CBT in reducing fatigue in PPS. The treatment protocol was developed by investigators with broad experience with CBT for chronic fatigue syndrome and fatigued cancer survivors, and customized to the specific aspects of fatigue in PPS.

Thirdly, the long-term follow-up, the broad arsenal of outcome measures on different domains of the ICF and the inclusion of a responsive evaluation and an economic evaluation gives this study a unique mixed methods design. Investigating patient perspectives and possible discrepancies between perspectives of patients and professionals will help to elaborate strong and weak points of both interventions, which in turn might explain specific results obtained in the study. Additionally, this mixed methods design will facilitate the decision making with respect to implementation of the results when the interventions turn out to be effective.

However, the study has also some limitations. The sample size was based on the detection of an approximately equal effect size of exercise training and CBT in comparison to usual care. Detecting differences in effectiveness between both interventions would require much larger sample sizes. Furthermore, the design of this study, will not give insight into the indicators that are helpful in decision making about tailoring interventions.

In conclusion, the results of this study will provide greater insight on evidence-based treatment options for PPS. Successful outcome from the study has the potential to result in reduced fatigue and improved activities and quality of life for patients with PPS. Results might lead to alterations of existing international guidelines.

## Abbreviations

PPS: Postpoliomyelitis Syndrome; CBT: cognitive behavioural therapy; GEE: generalized estimated equations; WHO: World Health Organization; HRQoL: health related quality of life; RCT: randomized controlled trial; AMC: Academic Medical Centre; UMCU: University Medical Centre Utrecht; UMCN: University Medical Centre Nijmegen; HRR: heart rate reserve; ICF: International Classification of Functioning; VE: ventilation; VO_2_: oxygen consumption; MVC: maximal voluntary contraction; VA: voluntary activation; QALYs: quality- adjusted life years; Bca: bias-corrected and accelerated; ICER: incremental cost-effectiveness ratio.

## Competing interests

The authors declare that they have no competing interests.

## Authors' contributions

FK is responsible for data collection, analysis and interpretation and wrote the manuscript. FN, AB, MdV, KG, GB and TA originated the idea of the study, developed the overall study design and obtained funding for the study. All authors read and approved the manuscript.

## Appendix 1: Overview of CBT intervention

### Perpetuating factors: dysfunctional cognitions with respect to the disease, pain and fatigue

A patient can have dysfunctional cognitions, for example unrealistic expectations on prognosis or difficulties with the acceptation of the consequences of PPS. More frequent are dysfunctional cognitions with respect to fatigue and/or pain. By non helping cognitions as 'I can't control the fatigue' and 'the fatigue is terrible', the patient catastrophizes about fatigue thereby enlarging the experience of fatigue. By means of cognitive restructuring (Socratic dialogue and prompting new self-talk) the patient learns to change these non helpful thoughts into more helpful one in daily life.

### Perpetuating factor: dysfunctional attention to fatigue and pain

Many patients are alert to symptoms of fatigue (or pain). Persons in their social environment ask the patient about the fatigue (or pain). Information and explanation will be given that the effect of attending to fatigue (or pain) sensations is increasing and intensifying these symptoms. By daily exercises the patient can learn how to shift attention away from fatigue (or pain). Also lessening the attention from others for these symptoms will be realized. The partner of the patient may be involved in this module.

### Perpetuating factor: deregulation of sleep

An irregular sleep-wake rhythm can perpetuate fatigue. To restore the biologic rhythm, patients will be encouraged to adhere to fixed bedtimes and wake-up times and will be discouraged from sleeping during the day or adapt fixed rest period(s).

### Perpetuating factors: deregulation of physical, social and mental activities

Polio survivors are known to have strong perseverance. From childhood on they were learned to deny their symptoms in order to achieve a normal life. Many of them have difficulties adapting their physical, social and mental activities to their decreasing abilities. First, these patients will be thought to pace or reduce the amount of activities. Next, graded increase of certain physical, social or mental activities will help the patient to reach step by step their set goals.

### Perpetuating factors: low social support and negative social interactions

Unrealistic expectations or non helpful cognitions about the social environment (for example 'why do I not get help from others?') can perpetuate fatigue. After explanation, the therapist will help to change non helpful cognitions and install more realistic expectations toward others in the patient's social environment. The partner or relevant others may be involved in this part of the therapy.

## Pre-publication history

The pre-publication history for this paper can be accessed here:

http://www.biomedcentral.com/1471-2377/10/8/prepub
